# Platelet mitochondrial respiration and coenzyme Q_10_ could be used as new diagnostic strategy for mitochondrial dysfunction in rheumatoid diseases

**DOI:** 10.1371/journal.pone.0256135

**Published:** 2021-09-28

**Authors:** Anna Gvozdjáková, Zuzana Sumbalová, Jarmila Kucharská, Monika Szamosová, Lubica Čápová, Zuzana Rausová, Oľga Vančová, Viliam Mojto, Peter Langsjoen, Patrik Palacka

**Affiliations:** 1 Pharmacobiochemical Laboratory of 3rd Department of Internal Medicine, Faculty of Medicine, Comenius University in Bratislava, Bratislava, Slovakia; 2 3rd Department of Internal Medicine, Faculty of Medicine, Comenius University in Bratislava, Bratislava, Slovakia; 3 Department of Rheumatology, University Hospital in Bratislava, Bratislava, Slovakia; 4 Private Cardiology Practice, Tyler, TX, United States of America; 5 2nd Department of Oncology, Faculty of Medicine, Comenius University in Bratislava, Bratislava, Slovakia; University of Windsor, CANADA

## Abstract

**Introduction:**

Rheumatoid arthritis (RA) is a chronic inflammatory autoimunne disorder affecting both small and large synovial joints, leading to their destruction. Platelet biomarkers are involved in inflammation in RA patients. Increased circulating platelet counts in RA patients may contribute to platelet hyperactivity and thrombosis. In this pilot study we evaluated platelet mitochondrial bioenergy function, CoQ_10_ levels and oxidative stress in RA patients.

**Methods:**

Twenty-one RA patients and 19 healthy volunteers participated in the study. High resolution respirometry (HRR) was used for analysis of platelet mitochondrial bioenergetics. CoQ_10_ was determined by HPLC method; TBARS were detected spectrophotometrically.

**Results:**

Slight dysfunction in platelet mitochondrial respiration and reduced platelet CoQ_10_ levels were observed in RA patients compared with normal controls.

**Conclusions:**

The observed decrease in platelet CoQ_10_ levels may lead to platelet mitochondrial dysfunction in RA diseases. Determination of platelet mitochondrial function and platelet CoQ_10_ levels could be used as new diagnostic strategies for mitochondrial bioenergetics in rheumatoid diseases.

## 1. Introduction

Rheumatoid arthritis (RA) is a chronic inflammatory autoimmune disorder affecting both small and large joints leading to their destruction. RA is frequently a progressive disease, with inflammation and tissue degradation in joints leading to painful deformity and immobility.

RA can also include effects on the cardiovascular system, cancer, and a wide spectrum of conditions such as depression, mental difficulties, fatique and physical disabilities. Clinical symptoms in early stage RA are characterized by swollen tender joints and morning stiffness. Indications of disease activity and progression are elevated levels of C-reactive protein (CRP) and an increased erythrocyte sedimentation rate [1]. Serum CRP levels in RA patients of around 3 mg/L are associated with an elevated risk of cardiovascular disease (CVD) and levels up to 10 mg/L are associated with a very high risk of CVD [2].

The chronic inflammatory state in insufficiently treated RA patients leads to a complex clinical picture with systemic manifestations noticed in lungs, muskuloskeletal system (cartilage destruction, bone erosion and loss range of motion) and hematologic abnormalities, such as anemia, leukopenia, and thrombocytopenia [3–5]. Tissue destruction erupts as synovitis, an inflammation of the joint capsule consisting of the synovial membrane and synovial fluid. Pro-inflammatory molecules such as cytokines contribute to the inflammation and platelet dysregulation in RA [6].

Genetic and environmental factors contribute to the clinical picture of RA. Genetic factors include the generation of autoreactive T and B cells with secondary viral or bacterial infections. Other factors, such as smoking, obesity, age, drugs, infections, lung disease and periodontal disease participate in RA [7, 8]. In early stage RA the production of reactive oxygen species (ROS) is elevated [9–13]. One of the main sources of ROS and adenosine triphosphate (ATP) production are the mitochondria. Several studies have reported mitochondrial dysfunction (not in platelets) and enhanced ROS production in rheumatoid arthritis [14–16].

In the pathophysiology of RA crutial role is played by the platelets, which contain only 5–8 mitochondria per platelet [17]. In the resting state platelets obtain ATP predominantly from glycolysis rather than oxidative phosphorylation (OXPHOS) [18]. Glycolysis produces lactate and generates two ATP molecules per one glucose molecule. OXPHOS produces 36 ATP molecules per one glucose molecule. Glycolysis produces ATP faster than OXPHOS [19].

An essential component of the mitochondrial respiratory chain for energy production is coenzyme Q_10_ (CoQ_10_) which also has antioxidant properties. Our previous study on the rat model of rheumatoid arthritis (adjuvant-induced arthritis) showed reduced mitochondrial oxidative phosphorylation in myocardial and skeletal muscle [20], increased markers of inflammation and decreased concentrations of coenzyme Q_9-OX_ in skeletal muscle tissue and mitochondria [21].

Platelet mitochondrial dysfunction has been documented in patients with several human diseases [18] including Alzheimer´s disease [22], depression [23], chronic kidney disease [24–26], patients after kidney transplantation [27]. Platelet mitochondrial dysfunction has also been observed in human ageing [28] and in winter time [29]. Data on platelet mitochondrial function in patients with RA are not available.

We performed studies to test the hypothesis that a deficit of CoQ_10_ and disturbance of platelet mitochondrial function occurs in RA patients. A deeper knowledge of RA effects on platelet mitochondrial activities and CoQ_10_ levels may contribute to the understanding of the pathobiochemical mechanisms of RA.

## 2. Materials and methods

### 2.1. Subjects

Twenty-one patients with confirmed RA participated in the study: 20 women and one man, aged from 38 to 79 years with a mean age of 61.2±28.6 years and a mean body mass index (BMI) of 25.8±1.08 kg.m^-2^. All chronic RA patients *(RA_ALL)* were treated with relevant conventional therapy (aceclofenac, vitamin D, methotrexate, plaquenil). From these patients, 5 of them were included in a subgroup *RA_CRP* (with high CRP from 9.50 mg/L to 11.99 mg/L) and 4 patients in a subgroup *RA_CVD* treated also with conventional therapy for cardiovascular diseases. The control group consisted of 19 human subjects (7 men and 12 women), aged from 56 to 81 years, with a mean age of 68.4±13.3 years, with a mean of BMI of 25.0±1.16 kg.m^-2^. RA patients on statins therapy were not included in the study.

The study was carried out according to the principles expressed in the Declaration of Helsinki, and the study protocol was approved by the Ethical Committee of the Academic Ladislav Dérer’s Hospital, Bratislava, Slovakia (1/0245/19, 27 June 2018). Written informed consent form was obtained from each subject before enrollment in the study.

### 2.2. Observed parameters

The following parameters were measured in a clinical biochemical laboratory using standard methods: blood hemoglobin, leucocytes and platelets count, serum c-reactive protein, serum creatinine, uric acid, AST (aspartate aminotransferase), ALT (alanine aminotransferase), GMT (gama-glutamyltransferase), total proteins (TP).

### 2.3. Coenzyme Q_10_ and oxidative stress

Coenzyme Q_10-TOTAL_ (ubiquinol+ubiquinone) in whole blood, plasma and isolated platelets were estimated using HPLC method with UV detection [30, 31]. For the oxidation of ubiquinol to ubiquinone, 100 μl of 1,4-benzoquinone (2 mg/1ml double distilled water–daily fresh) was added to 500 μl of blood or plasma and vortexed for 10 seconds [32]. After 10 minutes incubation at room temperature 2 ml of the mixture hexane/ethanol (5/2 v/v) was added, shaken for 5 minutes and centrifuged at 1 000 g for 5 minutes. The hexane layer was separated and extraction procedure was repeated with 1 ml of the extraction mixture. Collected organic layers were evaporated under nitrogen at 50°C. The residues were taken up in 99.9% ethanol and injected into a reverse phase HPLC column (SGX C18, 7 μm, Tessek Ltd). Elution was performed with methanol/acetonitrile/ethanol (6/2/2 v/v/v) at a flow rate 0.9 ml/min. The concentrations of CoQ_10-TOTAL_ were detected with an UV-detector at 275 nm, using an external standard (Sigma). Data were collected and processed with a CSW32 chromatographic station (DataApex Ltd). Concentrations of CoQ_10-TOTAL_ were calculated in μmol.l^-1^.

**Coenzyme Q**_**10-TOTAL**_**determination in platelets.** Isolated human platelets (approx 150–250 millions) were disintegrated with 500 μl of cold methanol [33]. Oxidation of ubiquinol to ubiquinone was performed with 1,4-benzoquinone as described for plasma extraction. The cell suspension was extracted with 2 ml hexane by shaking for 5 minutes. After centrifugation, organic layer was separated, evaporated and measured as described above. Concentrations of CoQ_10-TOTAL_ were calculated in pmol.10^-9^ cells.

A parameter of oxidative stress–thiobarbituric acid reactive substances (TBARS) was estimated by spectrophotometric method [34].

### 2.4. Platelet preparation

Blood samples were collected by venipuncture in two 9 mL K3EDTA (tripotassium ethylenediaminetetraacetic acid) tubes each day between 7:00 and 8:00 a.m. and transported at 25°C room temperature to the laboratory. For platelet (PLT) isolation, the tubes with blood were centrifuged at room temperature at 200×g for 10 min using swing-out rotor without breaking. Platelet-rich plasma (PRP) was transferred into a new plastic tube and mixed with 100 mM EGTA (ethylene glycol-bis(2-aminoethylether)-N,N,N´,N´-tetraacetic acid) to a final concentration of 10 mmol·L^−1^. The pellet after centrifugation at 1200×g, was washed with 4 mL of DPBS (Dulbecco’sPhosphate-Buffered saline) plus 10 mM EGTA and finally resuspended in 0.4 mL of the same solution. The PLT suspension was counted (10 times diluted) on hematological analyzer Mindray BC-2800 (Mindray, China) [35].

### 2.5. Platelet mitochondrial respiration and oxidative phosphorylation

**Fig 1:**
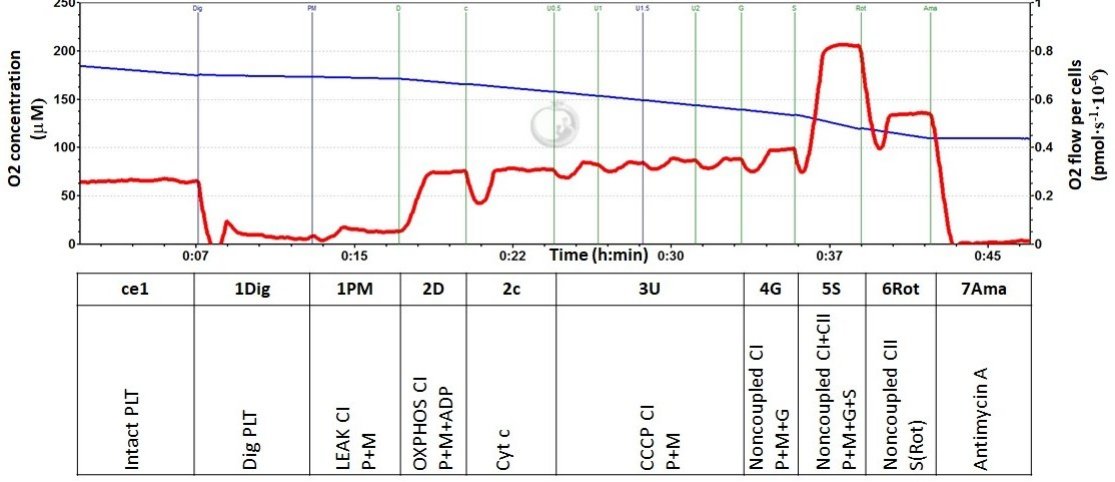
Respirometric analysis of mitochondrial function in human platelets. The trace from the measurement of platelet (PLT) respiration at 37°C in a respiration medium MiR05 and 20 mM creatine. The blue line represents oxygen concentration (μM), and the red trace represents oxygen consumption as flow per cells (pmol O_2_·s^−1^·10^−6^ cells). The modified substrate-uncoupler-inhibitor-titration (SUIT) reference protocol 1 (RP1) [38]. includes following steps: **ce1**: Oxygen consumption rate of intact PLT (routine respiration); **1Dig**: Respiration rate of mitochondria in PLT permeabilized with digitonin; **1PM:** Complex I-linked LEAK (State 4) respiration reflects the rate of mitochondrial respiration with exogenous substrates (pyruvate and malate); **2D**: Complex I-linked OXPHOS (State 3) after ADP addition reflects CI-linked ATP production; **2c**: Cytochrome c addition—a test for the integrity of outer mitochondrial membrane; **3U**: The rate after addition of an uncoupler CCCP represents maximal CI-linked oxidative capacity with substrates pyruvate and malate (uncoupled from OXPHOS). **4G:** Noncoupled Complex I-linked oxygen consumption after the addition of substrate glutamate; **5S:** Noncoupled Complex I- and Complex II-linked oxygen consumption after the addition of CII substrate succinate; **6Rot**: Noncoupled Complex II-linked oxygen consumption after the addition of rotenone—an inhibitor of Complex I; **7Ama**: Residual oxygen consumption (ROX) after the addition of antimycin A—an inhibitor of CIII represents respiration that is not associated with electron transfer pathways. This respiration is subtracted from all values for the determination of mitochondrial electron transfer pathways-related oxygen consumption.

Oxygen consumption in the intact and permeabilized platelets and the capacity of oxidative phosphorylation (OXPHOS) at Complex I were determined with the high-resolution respirometry (HRR) method. HRR is a sensitive technique to determine mitochondrial bioenergetic function in human platelets isolated from peripheral blood [36, 37]. For mitochondrial respirometric analysis, 200×10^6^ platelets were used in a 2 mL chamber of an O2k-Respirometer (Oroboros Instruments, Innsbruck, Austria). The respiration was measured at 37°C in a mitochondrial respiration medium, MiR05 and 20 mM creatine following SUIT protocol RP1 (Substrate-Uncoupler-Inhibitor-Titration). The data were collected with the DatLab software (Oroboros Instruments, Innsbruck, Austria) using a data recording interval of 2 s [36, 38]. A representative trace from the respirometric measurement is shown in Fig 1.

### 2.6. Data analysis

The results are reported as the mean ± standard error (sem). Statistical analyses were performed in Graph-Pad Prism 6 for Windows. One-way ANOVA with pairwise tests and Holm-Sidak’s correction for multiple comparisons were used for comparisons of groups with the control group. Differences are marked statistically significant where the respective adjusted p-values are less than 0.1. The levels of adjusted p-values vs control group are marked as follows: ****p<0.0001, ***p<0.001; **p<0.01 *p<0.05, ^**+**^p<0.1.

## 3. Results

### 3.1. Metabolic parameters of human volunteers and of RA patients

The concentration of *hemoglobin* in all groups of RA patients was similar to the control group. *The leucocyte count* was not significantly increased in RA_ALL patients (110.98%), in RA_CRP group (120.45%), in RA_CVD patients (101.20%) vs control group.

*The platelet count* in group of RA_ALL patients was significantly increased to 122.8% (p<0.05) vs control group; count of platelets in group RA_CRP was increased to 124.9% (p<0.1), the highest count of platelets was in RA_CVD subgroup to 140.8% (p<0.05) vs control data (Table 1 and Fig 3). *CRP*, the marker of inflammation, in RA_ALL patients was increased to 219.3% vs control data. The highest CRP was in group RA_CRP, increased to 613.3%, (p<0.0001) vs control data, in group RA_CVD patients CRP was similar to control group. *Creatinine* concentration in plasma was without significant differences between control and RA groups. Only in RA_CVD group was this parameter slightly decreased to in comparison with the control group. *Uric acid* concentration was significantly decreased in RA_ALL patients, vs control data (p<0.05), representing 84.55%. Liver enzymes showed various results. *AST* was in reference values, slightly influented by RA. In RA_ALL group of patients *AST* was not significantly increased to 113.48%, in RA_CRP decreased to 86.25%, in RA_CVD increased to 124.80% in comparison with control data. *ALT* activity in RA patients was not significantly increased from 120.07% (RA_ALL) to 168.46% (RA_CRP group). On the contrary, *GMT* acitivity was significantly reduced in RA_ALL patients (p<0.001) to 45.94% vs control data. In RA_CRP group of patients it was decreased to 52.20% (p<0.05), in RA_CVD patients to 60.85% (p<0.1). *Total proteins* were significantly increased in all RA groups in comparison with healthy human volunteers. In RA_ALL group (p<0.0001), in RA_CRP (p<0.0001), in RA_CVD group (p<0.0001), (Table 1).

**Table 1:** Metabolic parameters of human volunteers and of RA patients.

Groups	Control	RA_ALL	RA_CRP	RA_CVD
*Parameter*	(N = 12)	(N = 21)	(N = 5)	(N = 3)
Hgb (g/L)	140.5±2.3	136.2±2.1	136.0±4.3	140.0±4.0
Le (10x9/L	6.65±0.44	7.38±0.64	8.01±1.05	6.73±1.22
PLT (10x9/L)	219.2±9.4	269.3±13.1*	273.8±23.6^+^	308.7±65.9*
Creatinine (μmol/L)	73.3±5.0	70.2±3.2	76.6± 8.5	72.7±9.8
Uric acid (μmol/L)	308.7±17.0	261.1±11.0*	273.8±16.4	260.0±17.6
AST (μkat/L)	0.371±0.030	0.421±0.042	0.320±0.016	0.463±0.080
ALT (μkat/L)	0.297±0.018	0.335±0.043	0.470±0.142	0.250±0.020
GMT (μkat/L)	0.751±0.139	0.345±0.036***	0.392±0.121*	0.457±0.019^+^
CRP (mg/L)	1.50±0.20	3.29±0.79	9.20±1.12****	1.66±0.66
Total proteins (g/L)	28.7±4.7	69.6±0.8****	68.2±0.8****	71.6±0.8****

Hgb–hemoglobin; Le–leucocytes; PLT–platelets; AST–aspartate aminotranferase; ALT–alanine aminotransferase; GMT–gama-glutamyltransferase; CRP- c-reactive protein; Data of all groups are presented as mean±sem and statistically evaluated in comparison with control group.

****p<0.0001,

***p<0.001;

**p<0.01

*p<0.05, ^**+**^p<0.1 vs control group.

### 3.2. Platelet mitochondrial respiration and oxidative phosphorylation of control subjects and RA patients

In our trial we found slight differences in platelet mitochondrial oxygen consumption between groups of RA patients and control subjects. Differences between RA groups and control group statistically are evaluated by ANOVA test and in %.

**Fig 2:**
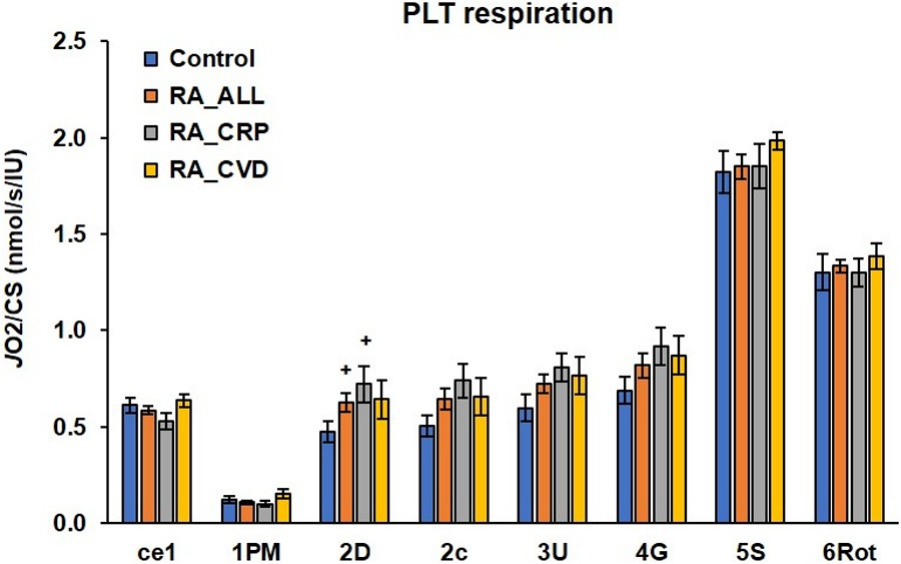
Platelet mitochondrial function in control subjects and groups of RA patients. The parameters of mitochondrial respiration in human platelets expressed as oxygen (O_2_) flux per mitochondrial marker–the activity of citrate synthase (CS). The bars show mean±sem. The names on x-axis represent steps in the SUIT protocol RP1 (see the legend for Fig 1). Control = control subjects; RA_ALL = all patients with rheumatoid arthritis; RA_CRP = rheumatic patients with high c-reactive protein concentration; RA_CVD = rheumatic patients with cardiovascular diseases; ^+^p<0.1.

Oxygen consumption in intact platelets (*ce1)* in RA_CRP group was decreased to 86.58% vs control group, in group RA_ALL and RA_CVD changed slightly vs control data. The rate of mitochondrial respiration with CI-linked substrates (*1PM–state 4*) in group of RA_ALL decreased to 87.90%; in group RA_CRP decreased to 81.45% vs control data and in group RA-CVD increased to 123.39% vs control data. CI-linked respiration coupled with ATP production *(2D* –CI-linked oxidative phosphorylation) was increased in group in RA_ALL to 132.21% (p<0.1); in RA_CRP to 152.00% (p<0.1); in RA_CVD to 135.16% vs control group. The respiration after addition of cytochrome c *(2c)* was increased in groups RA_ALL to 127.72%; in RA_CRP to 146.34%); in RA_CVD to 130.30%. Maximal oxidative capacity after uncoupler titration *(3U)* in group of RA_ALL was increased to 121.07%; in RA_CRP to 135.62%; in RA_CVD to 128.26% vs control group. After addition of exogenous substrate glutamte (*4G*) mitochondrial respiration was slightly increased in all RA groups, in RA_ALL to 118.89%; in RA_CRP to 133.72%; in RA_CVD to 126.60% vs control data. Noncoupled CI+CII mitochondrial respiration (*5S*) in all RA groups were similar to control group. Mitochondrial respiration of CII after inhibition of CI with rotenone (*6Ro*t) in RA groups was similar to control group (Fig 2 and Table 2).

**Table 2:** Platelet mitochondrial function in control subjects and groups of RA patients.

		*J*O2/CS [nmol/s/IU]		
Groups	Control	RA_ALL	RA_CRP	RA_CVD
*Parameter*	(N = 15)	(N = 21)	(N = 5)	(N = 3)
**ce1**	0.611±0.042	0.585±0.021	0.529±0.044	0.637±0.035
**1Dig**	0.045±0.013	0.038±0.006	0.041±0.015	0.053±0.018
**1PM**	0.124±0.017	0.109±0.008	0.101±0.015	0.153±0.027
**2D**	0.475±0.054	0.628±0.050^+^	0.722±0.094^+^	0.642±0.102
**2c**	0.505±0.054	0.645±0.052	0.739±0.089	0.658±0.096
**3U**	0.598±0.070	0.724±0.050	0.811±0.073	0.767±0.098
**4G**	0.688±0.070	0.818±0.063	0.920±0.098	0.871±0.099
**5S**	1.821±0.110	1.851±0.065	1. 852±0.115	1.984±0.044
**6Rot**	1.303±0.096	1.336±0.034	1.301±0.074	1.385±0.069

Names of the parameters are in Fig 1. Data of all groups are presented as mean±sem and statistically evaluated in comparison with control group; ^**+**^p<0.1 vs control group.

### 3.3. Coenzyme Q_10-TOTAL_ and TBARS in control subjects and in RA patients

Endogenous concentration of CoQ_10-TOTAL_ (ubiquinone + ubiquinol) *in platelets* of RA_ALL patients significantly decreased to 57.79% (p<0.0001), in group RA_CRP decreased to 59.42% (p<0.01) and in RA_CVD group to 87.02% (not significant) vs control group. Concentration of CoQ_10-TOTAL_ *in whole blood and in plasma* slightly decreased in RA_ALL and in RA_CRP groups, in RA_CVD decreased to 89.0% and 86.5% respectively vs control group. TBARS concentration was slightly increased in RA_ALL and in RA_CRP vs control data (Table 3).

**Table 3:** Endogenous coenzyme Q_10-TOTAL_ and TBARS in control subjects and in RA patients.

Groups	Control	RA_ALL	RA_CRP	RA_CVD
*Parameter*	(N = 18)	(N = 21)	(N = 5)	(N = 3)
**CoQ**_**10-TOTAL**_				
**Platelets** (pmol.10^-9^cells)	165.6.0±12.8	95.7±5.0****	98.4±4.9**	144.1±30.9
**Blood** (μmol.L^-1^)	0.271±0.031	0.289±0.028	0.254±0.063	0.241±0.047
**Plasma** (μmol.L^-1^)	0.409±0.039	0.408±0.029	0.383±0.049	0.354±0.064
**TBARS** (μmol.L^-1^)	4.71±0.14	4.90±0.15	5.21±0.28	4.58±0.23

^****^p<0.0001,

^**^p<0.01- statistically significant difference vs control group.

### 3.4. Platelet count and platelet coenzyme Q_10-TOTAL_ in control subjects and in groups of RA patients

**Fig 3:**
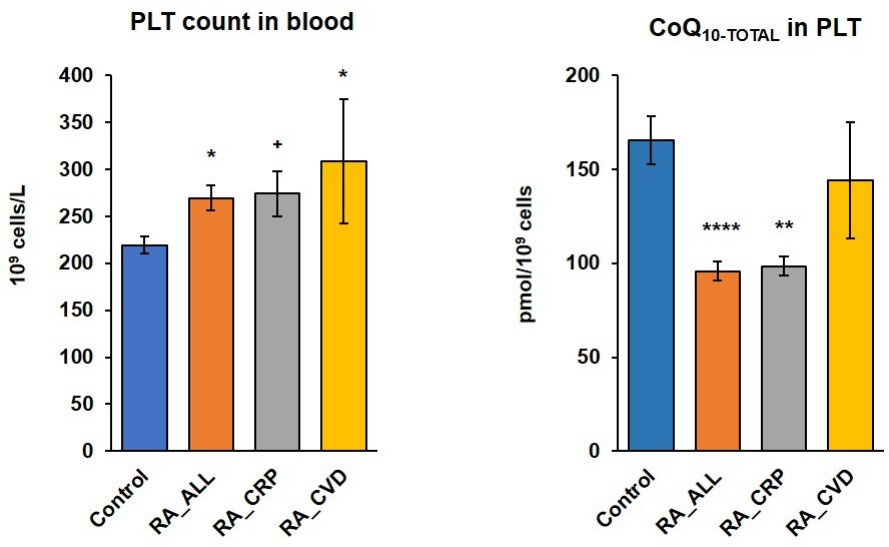
Platelet count and platelets coenzyme Q_10-TOTAL_ in control subjects and in groups of RA patients. CoQ_10-TOTAL_ (ubiquinol + ubiquinone); RA_ALL = all patients with rheumatoid arthritis; RA_CRP = rheumatic patients with high c-reactive protein concentration; RA_CVD = rheumatic patients with cardiovascular diseases; ^**+**^p<0.1*p<0.05; **p<0.01, ****p<0.0001.

Mean of *platelet count* and *platelet CoQ*_*10-TOTAL*_ concentration in control subjects and in RA patients shown in Table 1 and 3 and Fig 3.

## 4. Correlations between platelet CoQ_10-TOTAL_ and platelet mitochondrial respiration in control subjects and RA_ALL patients (Fig 4A–4D)

**Fig 4:**
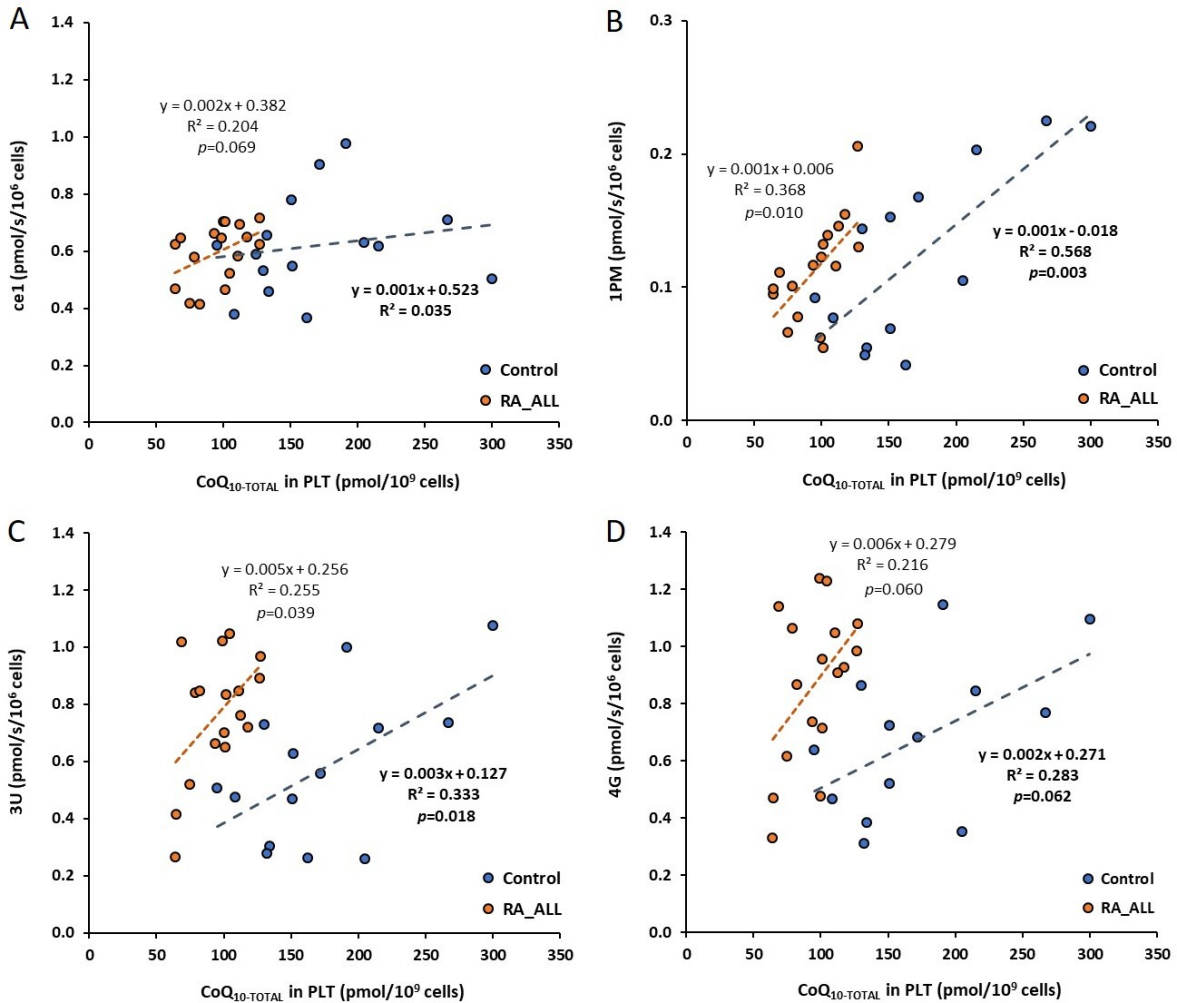
4A. Correlation between CoQ_10-TOTAL_ in Platelets and Respiration of Intact Platelets in Control Subjects and RA_ALL Patients. *ce1 = the rate of oxygen consumption in intact platelet; RA_ALL = all patients with RA; p = 0*.*069; CoQ*_*10-TOTAL*_ *= ubiquinol + ubiquinone; PLT = platelet*. 4B: Correlation between CoQ_10-TOTAL_ in Platelets and Mitochondrial Respiration in Permeabilized Platelets in Control Subjects and RA_ALL Patients. *1PM = the rate of oxygen consumption in mitochondrial of permeabilized platelets (State 4); RA_ALL = all patients with RA; p = 0*.*010*, *p = 0*.*003 = statistically significant; CoQ*_*10-TOTAL*_ *= ubiquinol + ubiquinone; PLT = platelet*. 4C: Correlation between CoQ_10-TOTAL_ in Platelets and 3U Platelets Mitochondrial Respiration in Control Subjects and RA_ALL Patients. *3U = the rate of oxygen consumption in platelet mitochondrial noncoupled respiration at CI; RA_ALL = all patients with RA; p = 0*.*039*, *in control group*: *p = 0*.*018; CoQ*_*10-TOTAL*_ *= ubiquinol + ubiquinone; PLT = platelet*. 4D: Correlation between CoQ_10-TOTAL_ in Platelets and 4G Platelets Mitochondrial Respiration in Control Subjects and RA_ALL Patients. *4G = the rate of oxygen consumption in platelets mitochondrial noncoupled CI after addition of substrate glutamate CI; RA_ALL = all patients with rheumatoid arthritis; p = 0*.*060; CoQ*_*10-TOTAL*_ *= ubiquinol + ubiquinone; PLT = platelet*.

Oxygen consumption by intact platelets (*ce1*) in RA_ALL patients positively correlated with CoQ_10-TOTAL_ concentration in platelets (p = 0.069), This correlation was not found in control subjects (Fig 4A). Oxygen consumption by mitochondria in permeabilized platelets (1PM) positively correlated with CoQ_10-TOTAL_ concentration in platelets of RA_ALL patients (p *=* 0.010) and control subjects (p = 0.003), (Fig 4B). We did not find significant correlation between CI-linked oxidative phosphorylation—ATP production (State 3) after ADP addition (2D) and CoQ_10-TOTAL_ concentration in platelets of control subjects or RA patients. The correlations between CoQ_10-TOTAL_ in platelets and 3U –platelet mitochondrial noncoupled respiration at CI in control subjects and RA_ALL patients were significant (p = 0.018, respectively p = 0.039), (Fig 4C). The correlation between platelets CoQ_10-TOTAL_ and noncoupled Complex I-linked oxygen consumption after addition of substrate glutamate (4G) was marginally significant in both control group (p = 0.062) and RA_ALL patients (p = 0.060), (Fig 4D). Noncoupled CI+CII-linked oxygen consumption after the addition of CII substrate succinate (5S), non-coupled CII-linked oxygen consumption after addition an inhibitor of Complex I (6Rot) did not correlate with CoQ_10-TOTAL_ concentration in platelets of control subjects and RA_ALL patients.

## 5. Discussion

This is the first study to examine platelet mitochondrial respiratory function and the relationship between platelet CoQ_10_ level and mitochondrial respiration in patients with rheumatoid arthritis (RA).

Platelets play a crutial role in pathophysiology of RA, they are small (2–4 μm) anucleate circulating fragments generated from the megakaryocytes in the bone marrow [39]. They are released from bone marrow into circulation, where they live for 7–10 days. Platelets are involved in wound healing, angiogenesis, immunoregulation, and inflammatory processes and they play an integral role in intracellular communication [40]. Their main function in the blood stream is a rapid binding to damaged blood vessels. In patients with RA activated PLTs release pro-inflammatory microparticles, which interact with white blood cells causing joint and systemic inflammation [41].

Platelet mitochondrial function is believed to reflect the mitochondrial health of the organism. A deeper knowledge of mitochondrial energy function in isolated platelets may contribute to a better understanding of the pathogenesis of RA and similar musculoskeletal diseases in humans [42, 43]. Individuals with mitochondrial disorders have comorbid conditions that may increase their risk for poor bone health [43].

In this trial several metabolic parameters were affected by RA: the count of leucocytes and platelets, CRP levels and total proteins were increased and GMT activity was reduced in RA patients (Table 1). Very high levels of CRP can be associated with very high risk of CVD [2]. Platelet count in RA patients was increased from 269.3 to 308.7x10^6^ platelets per mL of blood compared to control group at 219.2 x10^6^ platelets per mL of blood. Platelet counts in healthy adults are between 150 x10^6^ and 450 x10^6^ per mL of blood, and these counts are changed with diseases [39]. Increased platelet count in RA patients can contribute to platelet hyperactivity and to thrombosis [44].

Increased platelet mitochondrial mass was observed in patients with myeloproliferative neoplasms and increased tumor necrosis factor-α (TNF-α). Similar effects of TNF-alpha on platelets in ageing was shown recently. The authors suggest altered bioenergetics in platelets and higher levels of baseline ADP and ATP could be key players in platelet hyperactivity in old mice [45].

Platelets are metabolically active cell fragments with high energy consumption. At rest, platelets supply 60% of energy from glycolysis and 30–40% from mitochondrial oxidative phosphorylation [15]. ATP is essential for platelet function. Mitochondria are key to platelet function and survival [37]. Mitochondria are important for energy supply in all organisms and platelet mitochondria act as the main energy suppliers during thrombus formation. Platelets contain a small count of mitochondria [14], they are metabolically flexible, they are capable to adapt to different metabolic changes and can utilize glycolysis or fatty acid metabolism instead of mitochondrial ATP production via oxidative phosphorylation.

**Fig 5:**
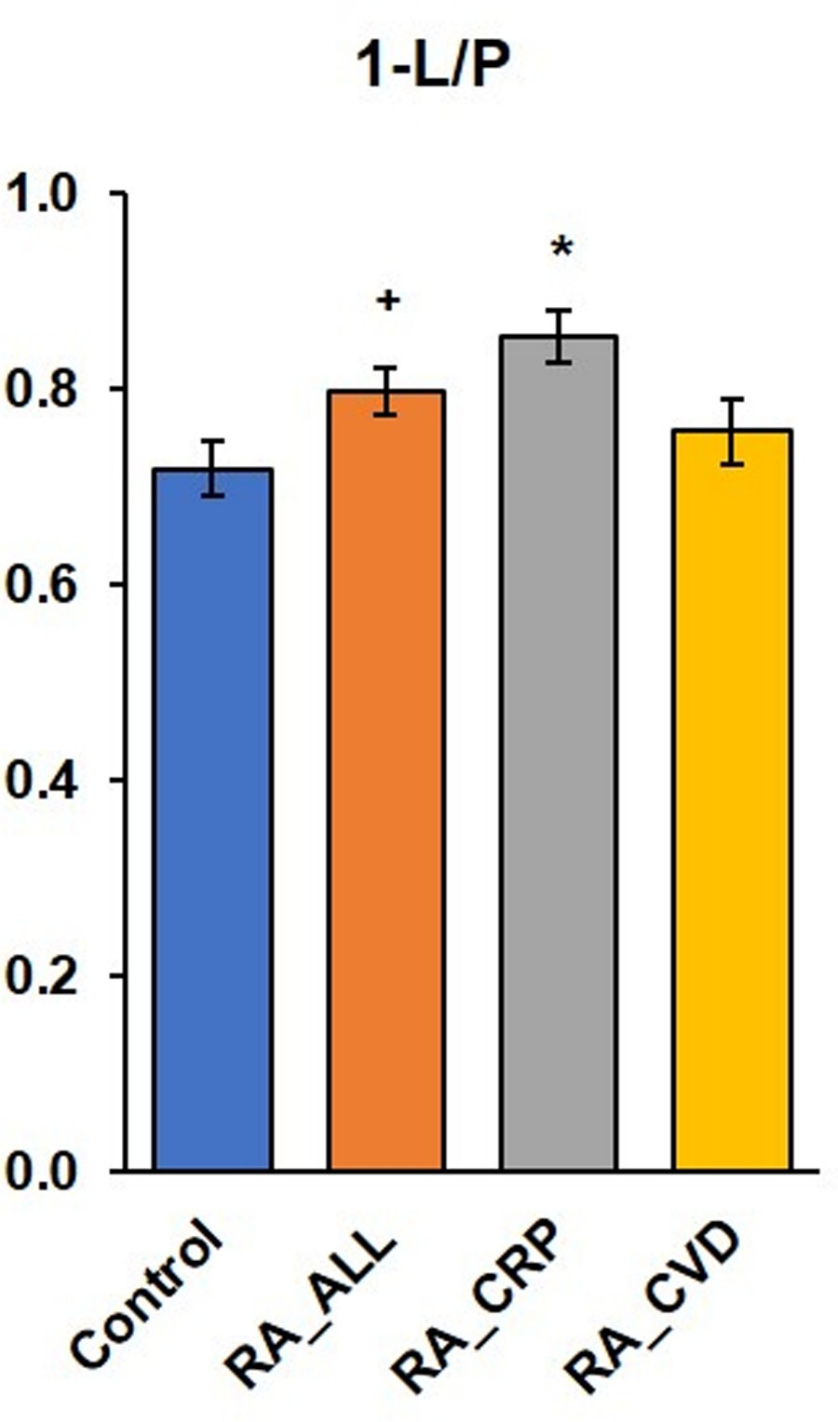
The OXPHOS-coupling efficiency in healthy control volunteers and groups of RA patients. RA_ALL = all patients with rheumatoic arthritis; RA_CRP = rheumatic patients with high c-reactive protein concentration; RA_CVD = rheumatic patients with cardiovascular diseases; ^+^p<0.5; *p<0.05 statistical significance vs control group.

The present study found slight differences in mitochondrial respiration of permeabilized platelets of RA patients in comparison with control human volunteers (Fig 2 and Table 2), the differences in platelet mitochondrial function between control and RA data we expressed in %, when control data were considered 100%. The lower oxygen consumption of intact platelets (ce1) in RA_CRP patients can indicate damaged platelet membrane permeability. In permeabilized platelets (1PM) in RA_CRP group, platelet mitochondrial respiration linked to Complex I (State 4) was decreased, which reflects decreased platelet mitochondrial oxygen consumption at Complex I. Mitochondrial oxygen uptake is slightly reduced and could be caused by increased anaerobic metabolism, reprogrammed platelet mitochondrial OXPHOS metabolism to glycolysis in rheumatoid arthritis. The increase of ADP-stimulated respiration at Complex I (State 3) in RA_ALL patients to 132.21%, in RA_CRP group of patients with higher CRP to 152.0% vs control data and the increased of OXPHOS-coupling efficiency could be platelet mitochondria adaptation to inflammation. OXPHOS-coupling efficiency parameter (1-L/P) [46] as respiratory control index, was higher in RA_ALL and RA_CRP groups in comparison with control group (Fig 5). Our results showed higher platelet counts and ATP production by OXPHOS in patients with rheumatic arthritis which may correspond with reported hyperreactivity of platelets in RA patients [41]. Upon platelet activation both glycolysis and OXPHOS are upregulated and platelets could receive relatively more energy from glycolysis or fatty acids metabolism than from OXPHOS in comparison with resting platelets [47]. In our study stimulated ATP production at CI, stimulated mitochondrial oxygen consumption in noncoupled state at complex I, in noncoupled states at CI+CII and at Complex II (after rotenone CI inhibition), support the hypothesis of platelet activation accompanied by mitochondrial energy reprogramming in RA patients.

Mitochondria are important not only for energy production, but also responsible for regulation of intracellular signalling through ROS. Mitochondrial function is impaired during uncontrolled ROS production, which has a critical role in the development and progression of diseases. Mitochondria initiate apoptosis through the release of cytochrome *c* into the cytoplasm. Released cytochrome *c* from mitochondria by inducing mitochondrial cristae remodeling was supported by inflammation as well as by increased oxidative stress [48]. An increase in the permeability of the membrane, a release of cytochrome *c* and a loss of mitochondrial membrane potential are signs of cell death [49]. Decreased level of CoQ_10_ in platelets and increased TBARS in RA patients with very high CRP concentration indicate increased oxidative stress that can contribute to the inflammatory processes (Table 3).

In the most common form of arthritis, osteoarthiritis, not only mitochondrial dysfunction but also mitochondrial DNA damage is involved [50]. Mitochondrial dysregulation and oxidative stress in patients with chronic kidney disease was reported [51] and in the last years disturbances in platelet mitochondrial function were documented in several human diseases [15].

Oxidative stress supported glycolytic metabolism may contribute to the acceleration of inflammatory mechanisms in RA [52]. Elevated levels of TBARS were found in several studies in the inflammed joints o RA patients [9–13].

CoQ_10_ plays a key role in mitochondrial energy production via OXPHOS, by way transfering electrons from Complex I and Complex II to Complex III along the respiratory chain in the inner mitochondrial membrane. Significantly reduced CoQ_10-TOTAL_ level in platelets together with oxidative stress in chronic inflammation in RA can lead to a deficit in platelet mitochondrial energy production, as well as to oxidative imbalance, which can enhance inflammation and tissue damage (Table 3 and Fig 3). Lipid peroxidation (TBARS) as an oxidative stress marker was increased in RA_ALL patients, in group of RA_CRP the values were highest. Decreased antioxidants level (ubiquinone + ubiquinol) and increased oxidative stress participate in dysbalance of oxido-redox potential of RA patients [11–13, 16, 52]. In other chronic inflammatory diseases, ankylosing spondylitis (AS), leading to joint disability, increased oxidative stress and decreased levels of total antioxidant status were observed in meta-analysis of 22 studies, including 931 AS patients [53]. Increased lipid peroxidation in RA patients may be a reason for platelet membrane damage (ce1) in RA patients. In a previous study we confirmed that high lipid peroxidation is a very important parameter in metastatic urothelial carcinoma patients, which was associated with poor survival [54].

Mitochondrial respiration significantly correlated with platelet CoQ_10-TOTAL_ concentration in RA patients as well as in control group (Fig 4B and 4C). Marginally significant correlations were found between platelet CoQ_10-TOTAL_ concentration and ce1 (Fig 4A) for RA_ALL patients and between CoQ_10-TOTAL_ concentration and 4G (Fig 4D) for both RA_ALL patients and control group. These results support mitochondrial energy reprogramming and flexibility of platelet mitochondrial function in RA patients.

CoQ_10-TOTAL_ level, measured in blood plasma may reflect mitochondrial bioenergy damage of various tissues. Based on our data in patients with RA the level of platelet CoQ_10_ seems to be crucial for oxygen comsumption by intact platelets or by mitochondria in platelets. Platelet CoQ_10-TOTAL_ level was positively correlated with platelet mitochondrial respiratory function in RA patients and it seems to be the most sensitive marker of platelet mitochondrial function in RA.

Decreased platelet CoQ_10-TOTAL_ level in chronic inflammatory RA diseases may lead to platelet mitochondrial dysfunction. Mitochondria are an important therapeutic target in osteoarthritis. Supplementary CoQ_10_ therapy can suppress the glycolytic pathway, decrease lactate production and upon glycolytic suppression the intracellular energy metabolism is reprogrammed toward mitochondrial OXPHOS [55]. CoQ_10_ supplementation in RA patients has beneficial effects on inflammatory cytokines (suppressed overproduction of TNF-α) and oxidative stress [56]. According to FL Crane, supplemental CoQ_10_ regulates outer mitochondrial membrane permeability, which depends on the VDAC (Voltage dependent Anion Channels) [57]. CoQ_10_ supplementation in RA patients can improve mitochondrial health and prevent or slow the progress of rheumatoid arthritis in humans.

## 6. Conclusion

This is the first study showed slight dysfunction of platelet mitochondrial respiration and reduced platelet CoQ_10_ level in patients with rheumatoid arthritis (RA). The most sensitive marker of platelet mitochondrial dysfunction in RA patients seems to be platelet CoQ_10-TOTAL_ levels which positively correlated with platelet mitochondrial respiratory function in RA patients. High resolution respirometry method for measurement of platelet mitochondrial function, and platelet coenzyme Q_10_ level determination could be used as a new diagnostic bioenergetic strategy in patients with rheumatoid arthritis. We conclude that decreased platelet CoQ_10-TOTAL_ level in chronic inflammatory RA diseases leads to platelet mitochondrial dysfunction. Reprogramming of platelet mitochondrial energy function could be possible by targeting supplementary therapy of RA patients with CoQ_10_. The results contribute to the understanding the pathobiochemical mechanisms of rheumatic diseases.

## Supporting information

S1 Data (XLSX)

## References

[1] Brzustewicz E, Henc I, Daca A, Szarecka M, Sochocka-Bykowska M, Witkowski J, et al. Autoantibodies, C-reactive protein, erythrocyte sedimentation rate and serum cytokine profiling in monitoring of early treatment. Cent Eur J Immunol 2017; 42: 259–268. doi: 10.5114/ceji.2017.70968 29204090 PMC5708207

[2] Graf J, Scherzer R, Grunfeld C, Imboden J. Levels of C-reactive protein associated with high and very high cardiovascular risk are prevalent in patients with rheumatoid arthritis. PloS ONE, 2009; 4/7: e6242. doi: 10.1371/journal.pone.0006242 19606218 PMC2707000

[3] Wolfe FL. Comparative usefulness of C-reactive protein and erythrocyte sedimentation rate in patients with rheumatoid arthritis. J Rheumatol 1997; 24: 1477–1485. 9263138

[4] Smolen JS, Aletaha D, McInnes IB. Rheumatoid arthritis. Lancet Lond Engl 2016; 388: 2023–2038. 10.1016/S0140-6736(16)30173-8 27156434

[5] Littlejohn EA, Monrad S. Early diagnosis and treatment of rheumatoid arthritis. Prim Care: Clin Off Pr 2018; 45: 237–255. 10.1016/j.pop.2018.02.010 29759122

[6] Aletaha D, Ramiro S. Diagnosis and Management of Rheumatoid Arthritis. JAMA 2018; 320: 1360–1372. doi: 10.1001/jama.2018.13103 30285183

[7] Deane KD, Demoruelle MK, Kelmenson LB, Kuhn KA, Norris JM, Holers VM. Genetic and environmental risk factors for rheumatoid arthritis. Best Pr Res Clin Rheumatol 2017; 31: 3–18. doi: 10.1016/j.berh.2017.08.003 29221595 PMC5726551

[8] Lin YU, Anzaghe M, Schulke S. Update on the pathomechanism, diagnosis, and treatment options for rheumatoid arthritis. Cells 2020; 9:880: doi: 10.3390/cells9040880 32260219 PMC7226834

[9] Taysi S, Polat F, Gul M, Sari RA, Bakan E. Lipid peroxidation, some extracellular anti-oxidants, and anti-oxidant enzymes in serum of patients with rheumatoid arthritis. Rheumatol Int 2002; 21: 200–204. doi: 10.1007/s00296-001-0163-x 11958437

[10] Stamp LK, Khalilova I, Tarr JM, Senthilmohan R, Turner R, Haigh RC, et al. Myeloperoxidase and oxidative stress in rheumatoid arthritis. Rheumatology (Oxford) 2012; 51: 1796–1803. doi: 10.1093/rheumatology/kes193 22814531

[11] Veselinovic M, Barudzic N, Vuletic M, Zivkovic V, Tomic-Lucic A, Djuric D, et al. Oxidative stress in rheumatoid arthritis patients: relationship to disease activity. Mol Cell Biochem 2014; 391: 225–232. doi: 10.1007/s11010-014-2006-6 24610042

[12] Garcia-González A, Gaxiola-Robles R, Zenteno-Savín T. Oxidative stress in patients with rheumatoid arthritis. Rev Invest Clin 2015; 67: 46–53. 25857584

[13] Mateen S, Moin S, Khan AG, Zafar A, Fatima N. Increased reactive oxidative species formation and oxidative stress in rheumatoid arthritis. PLoS ONEdoi: 10.1371/journal.pone.0152925 April 4, 2016. 27043143 PMC4820274

[14] Fearon U, Canavan M, Biniecka M, Vaele DJ. Hypoxia, mitochondrial dysfunction and synovial invasiveness in rheumatoid arthritis. Nat Rev Rheumatol 2016; 12: 385–397. 10.1038/nrrheum.2016.69 27225300

[15] Panga V, Kallor AA, Nair A, Harshan S, Raghunathan S. Mitochondrial dysfunction in rheumatoid arthritis: A comprehensive analysis by integrating gene expression, protein-protein interactions and gene ontology data. PLoS ONE; 2019; 14(11): e0224632. 10.1371/journal.pone.0224632 31703070 PMC6839853

[16] Sá da Fonseca LJ, Nunes-Souza V, Fonseca Goulart MO, Rabelo LA. Oxidative stress in rheumatoid arthritis: What the future might hold regarding novel biomarkers and add-on therapies. Oxid Med Cell Longevity, Volume 2019, Article ID 7536805, 16 pages. 10.1155/2019/7536805 PMC6942903 31934269

[17] Hayashi T, Tanaka S, Hori Y, Hirayama F, Sato EF, Inoue M. Role of mitochondria in the maintenance of platelet function during in vitro storage. Transf Med 2011; 21: 166–74. doi: 10.1111/j.1365-3148.2010.01065.x 21208306

[18] Wang L, Wu Q, Fan Z, Xie R, Wang Z, Lu Y. Platelet mitochondrial dysfunction and the correlation with human diseases. Biochem Soc Trans 2017; 45:1213. doi: 10.1042/BST20170291 29054925

[19] Pfeiffer T, Schuster S, Bonhoeffer S. Cooperation and competition in the evolution of ATP-producing pathways. Science 2001; 292: 504–507 doi: 10.1126/science.1058079 11283355

[20] Bauerová K, Kucharská J, Poništ S, Gvozdjáková A. Coenzyme Q10 supplementation in adjuvant arthritis (experimental model). In: Gvozdjáková A., Mitochondrial Medicine, Springer, Netherlands, 2008: 340–342.

[21] Bauerova K, Kucharska J, Ponist S, Slovak L, Svik K, Jakus V, et al. The role of endogenous antioxidants in the treatment of experimental arthritis. Chapter. In: Antioxidants, IntechOpen, Book, 2019: 10.5772/intechopen.85568

[22] Fišar Z, Jirák R, Zvěřová M, Setnička V, Habrtová L, Hroudová J, et al. Plasma amyloid beta levels and platelet mitochondrial respiration in patients with Alzheimer´s disease. Clin Biochemistry, 10.1016/j.clinbiochem.2019.04.003 30954436

[23] Hroudová J, Fišar Z, Kitzlerová E, Zvěřová M, Raboch J.: Mitochondrial respiration in blood platelets of depressive patients. Mitochondrion 2013; 13: 795–800. doi: 10.1016/j.mito.2013.05.005 23688905

[24] Sumbalová Z, Gvozdjáková A, Kucharská J, Rausová Z, Vančová O, Kuzmiaková Z, et al. Platelet mitochondrial function, coenzyme Q10, and oxidative stress in patients with chronic kidney diseases. MiP2019/MitoEAGLE 2019: 35–36. 10.3390/diagnostics10030176 PMC7151406 32210203

[25] Gvozdjáková A, Kucharská J, Sumbalová Z, Rausová Z, Chládeková A, Komlosi M, et al. The importance of coenzyme Q10 and its ratio to cholesterol in the progress of chronic kidney diseases linked to non-communicable diseases. Bratisl Med J 2020; 121/10. 693–699. doi: 10.4149/BLL_2020_113 32955899

[26] Gvozdjáková A, Sumbalová Z, Kucharská J, Komlósi M, Rausová Z, Vančová O, et al. Platelet mitochondrial respiration, endogenous coenzyme Q10 and oxidative stress in patients with chronic kidney disease. Diagnostics 2020; 10, 176; doi: 10.3390/diagnostics10030176 32210203 PMC7151406

[27] Gvozdjáková A, Sumbalová Z, Kucharská J, Chládeková A, Rausová Z, Vančová O, et al. Platelet mitochondrial bioenergetic analysis in patients with nephropathies and non-communicable diseases: a new method. Bratisl Med J 2019; 120/9: 630–635. doi: 10.4149/BLL_2019_104 31475544

[28] Gvozdjáková A, Sumbalová Z, Kucharská J, Chládeková A, Rausová Z, Vančová O, et al. Platelets mitochondrial function depends on coenzyme Q10 concentration in human young, not in elderly subjects. J Nutritional Therapeutics 2018; 7: 67–76.

[29] Gvozdjáková A, Kucharská J, Sumbalová Z, Nemec M, Chládeková A, Vančová O, et al. Platelet mitochondrial function depends on CoQ10 concentration in winter, not in spring season. Gen Physiol Biophys 2019; 38: 325–334. doi: 10.4149/gpb_2019012 31241044

[30] Lang J K, Gohil K, Packer L. Simultaneous determination of tocopherols, ubiquinols, and ubiquinones in blood, plasma, tissue homogenates, and subcellular fractions. Analyt. Biochem 1986, 157:106–116. 10.1016/0003-2697(86)90203-4 3766953

[31] Kucharská J, Gvozdjáková A, Mizera S, Braunová Z, Schreinerová Z, Schrameková E, et al. Participation of coenzyme Q10 in the rejection development of the transplanted heart. Physiol Res 1998; 47: 399–404. 10453746

[32] Mosca F, Fattorini D, Bompadre S, Littarru GP. Assay of coenzyme Q10 in plasma by a single dilution step. Anal. Biochem. 2002; 305: 49–54 10.1006/abio.2002.5653 12018945

[33] Niklowitz P, Menke T, Andler WM, Okun JG. Simultaneous analysis of coenzyme Q10 in plasma, erythrocytes and platelets: comparison of the antioxidant level in blood cells and their enviroment in healthy children and after oral supplementation in adults. Clin Chim Acta 2004; 342: 219–226. doi: 10.1016/j.cccn.2003.12.020 15026284

[34] Janero DR, Bughardt B. Thiobarbituric acid-reactive malondialdehyd formation during suproxide-dependent, iron-catalyzed lipid peroxidation: influence of peroxidation conditions. Lipids 1989; 24: 125–131 10.1007/BF02535249 2547130

[35] Sumbalová Z, Droescher S, Hiller E, Chang S, Garcia L, Calabria E, et al. O2k-Protocols: Isolation of peripheral blood mononuclear cells and platelets from human blood for HRR. Mitochondr Physiol Netw 2018; 17: 1–16.

[36] Pesta D, Gnaiger E. High-resolution respirometry: OXPHOS protocols for human cells and permeabilized fibers from small biopsies of human muscle. Methods Mol. Biol. 2012; 810: 25–58. 21. doi: 10.1007/978-1-61779-382-0_3 22057559

[37] Sjövall F, Ehinger JK, Marelsson SE, Morota S, Frostner EA, Uchino H, et al. Mitochondrial respiration in human viable platelets–methodology and influence of gender, age and storage. Mitochondrion 2013; 13: 7–14. doi: 10.1016/j.mito.2012.11.001 23164798

[38] Doerrier C, Sumbalová Z, Krumschnabel G, Hiller E, Gnaiger E. SUIT reference protocol for OXPHOS analysis by high-resolution respirometry. Mitochondr. Physiol. Netw. 2016; 21: 1–12.

[39] Ghoshal K, Bhattacharyya M: Overview of platelet physiology: its hemostatic and nonhemostatic role in disease pathogenesis. Scientific World Journal 2014; 2014:781857. doi: 10.1155/2014/781857 24729754 PMC3960550

[40] Melchinger H, Jain K, Tyagi T, Hwa J. Role platelet mitochondria: Life in a nucleus-free zone. Frontiers in Cardiovascular Medicine 2019; doi: 10.3389/fcvm.2019.00153 31737646 PMC6828734

[41] Harifi G, Sibilia J. Pathogenic role of platelets in rheumatoid arthritis and systemic autoimmune diseases: perspectives and therapeutic aspects. Saudi Med J 2016; 37(4): 354–360. doi: 10.15537/smj.2016.4.14768 27052277 PMC4852012

[42] Roberta C, Alberto F. Mitochondriopathies and bon health. J Trends in Biomedical Research 2018; 1/1: 1–7.

[43] Gandhi SS, Muraresku S, McCormick EM, Falk MJ, McCormack SE. Risk factors for poor bone health in primary mitochondrial disease. J Inherit Metab Dis 2017; 40/5: 673–683. doi: 10.1007/s10545-017-0046-2 28451918 PMC5659975

[44] Lee HC, Yin PH, Lu CY, Chi CW, Wei YH. Increase of mitochondria and mitochondrial DNA in response to oxidative stress in human cells. Biochem J 2000; 348: 425–432. 10816438 PMC1221082

[45] Dazivon-Castillo P, McMahon B, Aquilla S, Bark D, Ashworth K, et al: TNF-α-driven inflammation and mitochondrial dysfunction define the platelet hyperactivity of ageing. Blood 2019; 134/9: 727–740. doi: 10.1182/blood.2019000200 31311815 PMC6716075

[46] https://wiki.oroboros.at/index.php/OXPHOS-coupling_efficiency_P-L

[47] Nayak MK, Dhanesha N, Doddapattar P, Rodriguez O, Sonkar VK, Dayal S, et al. Dichloroacetate, an inhibitor of pyruvate dehydrogenase kinases, inhibits platelet aggregation and arterial thrombosis. Blood Adv 2018; 2/15: 2029–2038. doi: 10.1182/bloodadvances.2018022392 30108111 PMC6093723

[48] Otera G, Miyata N, Kuge O, Mihara K. Drp1-dependent mitochondrial fission via MiD49/51 is essential for apoptotic cristae remodeling. Journal Cell Biol 2016; 212/5: 531–544. doi: 10.1083/jcb.201508099 26903540 PMC4772499

[49] Maneiro E, Martín MA, de Andres MC, López-Armanda MJ, Fernadéz-Suerio JL, Hoyo P, et al. Mitochondrial respiratory activity is altered in osteoarthritic human articular chondrocytes. Arthritis & Rheumatism 2003; 48/3: 700–708. doi: 10.1002/art.10837 12632423

[50] Rego-Pérez I, Durán-Sotuela A, Ramos-Louro P, Blanco FJ. Mitochondrial genetics and epigenetics in osteoarthritis. Frontiers in Genetics 2020; Volume 10, Article 1336. doi: 10.3389/fgene.2019.01335 32010192 PMC6978735

[51] Granata S, Zaza G, Simone S, Villani G, Latorre D, Pontrelli P, et al. Mitochondrial dysregulation and oxidative stress in patients with chronic kidney disease. BMC Genom 2009; 10: 388. doi: 10.1186/1471-2164-10-388 19698090 PMC2737002

[52] Balogh E, Veale J, McGarry T, Orr C, Szekanecz Z, Ng C, et al. Oxidative stress impairs energy metabolism in primary cells and synovial tissue of patients with rheumatoid arthritis. Arthritis Research & Therapy 2018; 10:95: 10.1186/s13075-018-1592-1 29843785 PMC5972404

[53] Li J, Liu S, Cui Y. Oxidative and antioxidative stress liked biomarkers in ankylosing spondylitis: A systematic review and meta-analysis. Oxidative Med Cell Longevity, Volume 2020; Article ID 4759451, 10 pages.

[54] Slopovsky J, Kucharska J, Obertova J, Mego M, Kalavska K, Cingelova S, et al. Plasma thiobarbituric acid reactive substances predicts survival chemotherapy naive patients with metastatic urothelial carcinoma. Translational Oncology 2021; 14: 100890; 10.1016/j.tranon.2020.100890 33059122 PMC7566937

[55] Shiratori R, Furuichi K, Yamaguchi M, Miyazaki N, Aoki H, Chibana H, et al. Glycolytic suppression dramatically changes the intracellular metabolic profile of multiple cancer cell lines in a mitochondrial metabolism-dependent manner. Scientific Reports 2019; 9: 18699; 10.1038/s41598-019-55296-3 31822748 PMC6904735

[56] Abdollahzad H, Aghdashi MA, Jafarabadi MA, Alipour B. Effect of coenzyme Q10 supplementation on inflammatory cytokines (TNF-α, IL-6) and oxidative stress in rheumatoid arthritis patients: A randomized controlled trial. Arch Med Res 2015; 46: 527–533. doi: 10.1016/j.arcmed.2015.08.006 26342738

[57] Gvozdjáková A, Kucharská J, Ostatníková D, Babinská K, Nakládal D, Crane FL. Ubiquinol improves symptoms in children with autism. Oxid Med Cell Longevity 2014; Article ID 798 957, 6 pages, 10.1155/2014/978957 24707344 PMC3953391

